# PVA Hydrogels Supplemented with PLA Mesh for Tissue Regeneration Scaffold

**DOI:** 10.3390/gels10060364

**Published:** 2024-05-25

**Authors:** Young-Ho Seo, Jae-Man Lee, Sun-Young Park, Myung-Hoo Kim, Seon-Beom Kim, Tae-Hwan Oh

**Affiliations:** 1 Department of Advanced Organic Materials Engineering, Graduate School, Yeungnam University, Gyeongsan 38541, Republic of Korea; seoyh@pusan.ac.kr; 2 Institute for Future Earth, Pusan National University, Busan 46241, Republic of Korea; psy731@pusan.ac.kr (S.-Y.P.); mhkim18@pusan.ac.kr (M.-H.K.); sbkim@pusan.ac.kr (S.-B.K.); 3 School of Chemical Engineering, Yeungnam University, Gyeongsan 38541, Republic of Korea; ljm9390@naver.com; 4 Department of Animal Science, Pusan National University, Miryang 50463, Republic of Korea; 5 Department of Food Science & Technology, Pusan National University, Miryang 50463, Republic of Korea

**Keywords:** polyvinyl alcohol, polylactic acid, mesh fabric, scaffold, cell proliferation

## Abstract

This study examined the tensile strength and biocompatibility properties of polyvinyl alcohol (PVA) hydrogel tissue regeneration scaffolds with polylactic acid (PLA) mesh fabric added as reinforcement, with a focus on the impact of heat treatment temperature and the number of layers of the PLA mesh fabric. The hydrogel scaffolds were prepared using a freeze–thaw method to create PVA hydrogel, with the PLA mesh fabric placed inside the hydrogel. The swelling ratio of the PVA/PLA hydrogel scaffolds decreased with increasing layer number and heat treatment temperature of the PLA mesh. The gel strength was highest when five layers of PLA mesh fabric were added, heat-treated at 120 °C, and confirmed to be properly placed inside the hydrogel by SEM images. The MTT assay and DAPI staining using HaCaT cells demonstrated that the cell proliferation was uninterrupted throughout the experimental period, confirming the biocompatibility of the scaffold. Therefore, we confirmed the possibility of using PLA mesh fabric as a reinforcement for PVA hydrogel to improve the strength of scaffolds for tissue regeneration, and we confirmed the potential of PLA mesh fabric as a reinforcement for various biomaterials.

## 1. Introduction

Tissue engineering, the implantation of artificial materials, is one of the most researched fields of science and is used in regenerative medicine. Tissue engineering has four main objectives: restoring, replacing, maintaining, or enhancing the function of different types of biological tissues [1,2]. Poly(vinyl alcohol) (PVA) is a highly versatile synthetic polymer that forms hydrogen bonds between and within molecules. It is used in a wide range of biomedical applications, including as a scaffold for cell culture, an embolic agent, and wound dressing. Its excellent biocompatibility makes it an ideal biomaterial [3]. PVA hydrogels are highly biocompatible and behave like living tissues when they absorb large amounts of water and form three-dimensional scaffolds through physical and chemical crosslinking. They are a key biomaterial for drug delivery systems and tissue engineering [4]. However, the mechanical properties of PVA hydrogels limit their application as biomaterials, requiring structural improvements. Related research is underway to improve their mechanical properties. In particular, studies have reported that the physical structure and mechanical strength of PVA hydrogel scaffolds influence cell growth behavior [5]. PVA exhibits strong hydrophilicity due to the large number of hydroxyl groups in the polymer chain. This allows water molecules to enter the polymer chain in a humid environment, causing it to swell and reducing its barrier properties and mechanical properties [6]. This can be overcome by using chemical crosslinking agents to improve resistance to water through the crosslinking reaction of PVA. Chemical crosslinkers for PVA include glutaraldehyde [7,8,9,10], glyoxal [11], hexamethylene diisocyanate [12], and boric acid [13,14], among others. Among these, aldehyde-based crosslinkers such as glutaraldehyde and formaldehyde are especially effective. However, chemical crosslinking has the disadvantage of being toxic to cells, making it difficult to use in food and pharmaceuticals, and causing environmental pollution [15]. Heat treatment is sometimes used to increase the crystallinity of PVA to increase its stability in water. However, in this case, physical crosslinking by aggregation can interfere with the crystallization of PVA, reducing its crystallinity [16]. This reduces its mechanical strength. In addition to chemical crosslinking, using the freeze–thaw method with PVA hydrogels results in the physical crosslinking of the polymers in the hydrogel, which improves mechanical properties and loses water-soluble properties [17].

Poly(lactic acid) (PLA) is a natural-based biopolymer that is actively used in disposable plastics and medical applications. It is a thermoplastic aliphatic polyester [18] that degrades in vivo, is characterized by biological stability, and has been approved by the U.S. Food and Drug Administration for use in the human body. The U.S. Food and Drug Administration (FDA) has approved PLA as a polymer for use in the human body due to its excellent biocompatibility and low toxicity [19,20,21]. These properties have led to its use in a variety of applications [22,23], including orthopedic screws, pins, and plates. Controlled drug delivery devices, scaffolds for bone regeneration, and its excellent mechanical strength have made it a widely used scaffold material [24,25]. Another example of a disposable textile product that utilizes biodegradable plastics is the tea bag, which is made from PLA monofilament. PLA tea bags are made by extruding PLA into monofilaments by melt-spinning and then making it into a mesh fabric, where tea powder is placed inside the mesh fabric with certain pores. The tea ingredients are extracted through the pores of the mesh fabric. In our previous work, we demonstrated the scaffold applicability of PLA mesh fabric using PLA mesh fabric alone [26], but to the best of our knowledge, this is the first time that reinforcement of PLA mesh fabric has been applied to PVA hydrogel.

In this study, we prepared a PVA hydrogel and added PLA mesh fabric inside it as reinforcement to improve the mechanical strength of the scaffold for tissue regeneration and its effect on cell proliferation.

## 2. Results and Discussion

### 2.1. Gel Content and Swelling Ratio

**Figure 1:**
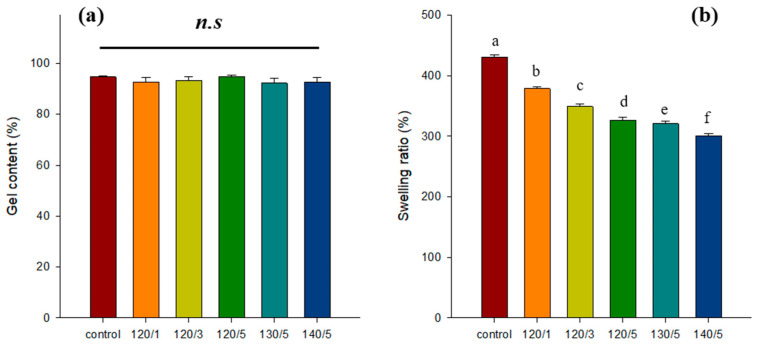
Gel content (**a**) and swelling ratio (**b**) of PVA/PLA hydrogels for different heat-treated temperatures and number of PLA mesh layers. Different letters denote statistically significant differences (*p* < 0.05) as determined by one-way ANOVA followed by Duncan’s multiple-range test.(*n.s* = No Significance).

Figure 1 shows the gelation content and swelling ratio of PVA/PLA hydrogels as a function of PLA heat-treated temperature and number of layers. The control sample consisted solely of PVA hydrogel without any PLA reinforcement. The graph clearly demonstrates that the gelation content does not have a significant difference depending on the PLA heat-treated temperature and the number of layers. PLA reinforcement did not significantly affect the gel formation of PVA using the freeze–thaw method. However, the swelling ratio decreased from 430 to 378, 348, 326, 320, and 300% as the heat-treated temperature and number of layers of PLA increased. This is caused by spatial limitations of the PVA polymer in the mold due to PLA insertion. This means that as the number of PLA layers increases, there is less space for the PVA polymer to enter the mold. The higher PLA heat-treated temperature fills the pores in the mesh fabric, making it more difficult for the PVA polymer to penetrate the PLA, resulting in a smaller amount of PVA polymer forming in the mold.

### 2.2. Gel Strength

**Figure 2:**
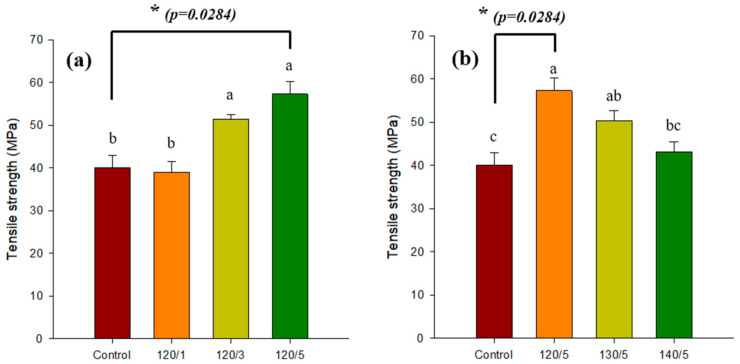
Tensile strength of PVA/PLA hydrogels: (**a**) for different numbers of PLA mesh layers at 120 °C; (**b**) for different heat-treated temperatures of 5 layers of PLA mesh. The data are presented as mean ± standard deviation. Significant differences between treatments were identified using a two-sample *t*-test (* *p* < 0.05) and are denoted by asterisks. Post hoc analysis using one-way ANOVA and Duncan’s multiple-range test revealed significant differences (*p* < 0.05) between groups, which are indicated by different letters.

Figure 2 shows the tensile strength results of the PVA/PLA hydrogel scaffold as a function of the number of layers of PLA mesh fabric and the heat-treated temperature. Figure 2a demonstrates that as the number of layers increased, the tensile strength of the PLA mesh fabric tended to increase. The results are unambiguous. Figure 2b shows the tensile strength results with the number of layers fixed and the heat-treated temperature increasing. The tensile strength tended to decrease as the heat-treated temperature increased. These results confirm that five layers of PLA mesh fabric heat-treated at 120 °C with PVA hydrogel as reinforcement is the optimal condition. This is consistent with the decrease in the swelling ratio. The decrease in strength is due to the high heat-treated temperature filling the pores of the PLA mesh fabric, making it difficult for the PVA polymer solution to penetrate and completely envelop the PLA mesh fabric.

### 2.3. FT-IR

**Figure 3:**
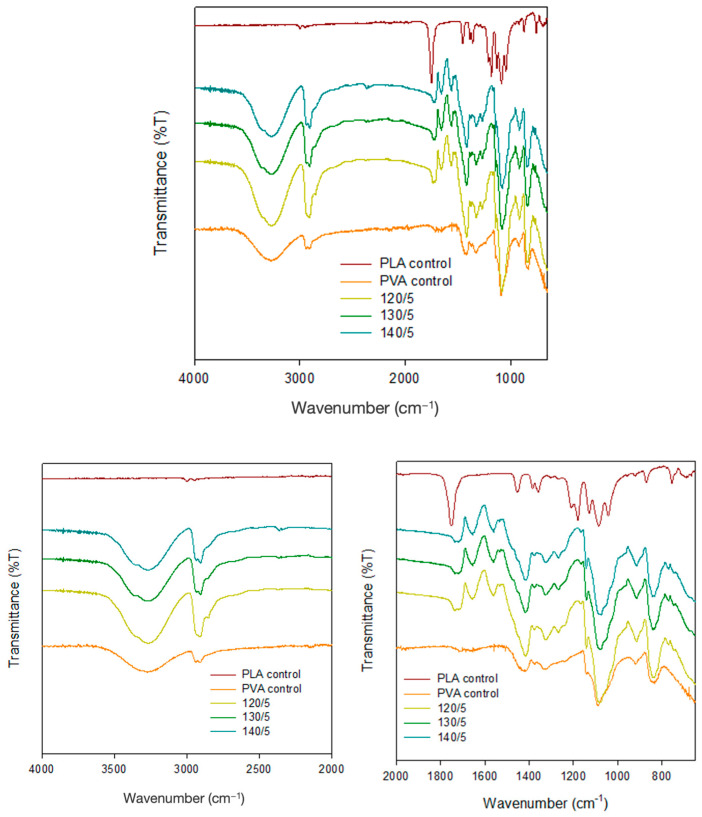
FT-IR spectra of PVA/PLA (5 layers) scaffold for different heat-treated temperatures.

FT-IR analysis confirmed the incorporation of PLA mesh in PVA hydrogel. Figure 3 shows the peaks corresponding to the -OH stretching vibration of PVA in the range of 3000–3600 cm^−1^ and the C-H stretching vibration in the range of 2800–3000 cm^−1^. Furthermore, in samples containing PLA mesh, a distinct peak corresponding to the C=O stretching vibration was observed at 1735 cm^−1^.

### 2.4. DSC

**Figure 4:**
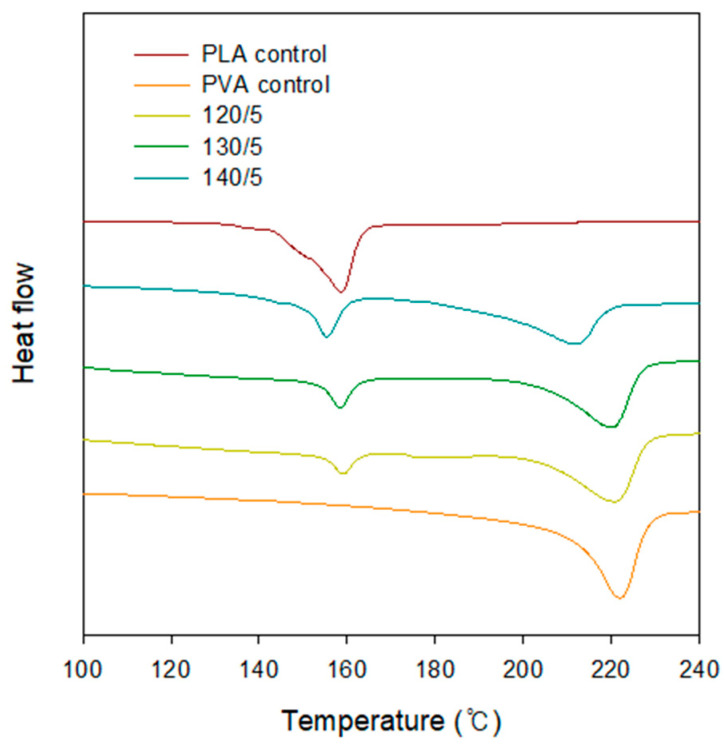
DSC curves of PVA/PLA (5 layers) hydrogels for different heat-treated temperatures.

Figure 4 shows the DSC measurement results of the PVA/PLA scaffold. The measured melting temperatures (T_m_) are shown in Table 1. The scaffold reinforced with PLA mesh fabric exhibited two distinct melting peaks: the PLA peak at T_1_ and the PVA peak at T_2_. The melting point of the PLA mesh fabric did not change significantly with the heat treatment temperature. However, the sample heated at 140 °C had a lower melting point, by 10 °C. This phenomenon is believed to be the result of incomplete crystal formation when PLA mesh fabric is heated at high temperatures.

### 2.5. SEM

**Figure 5:**
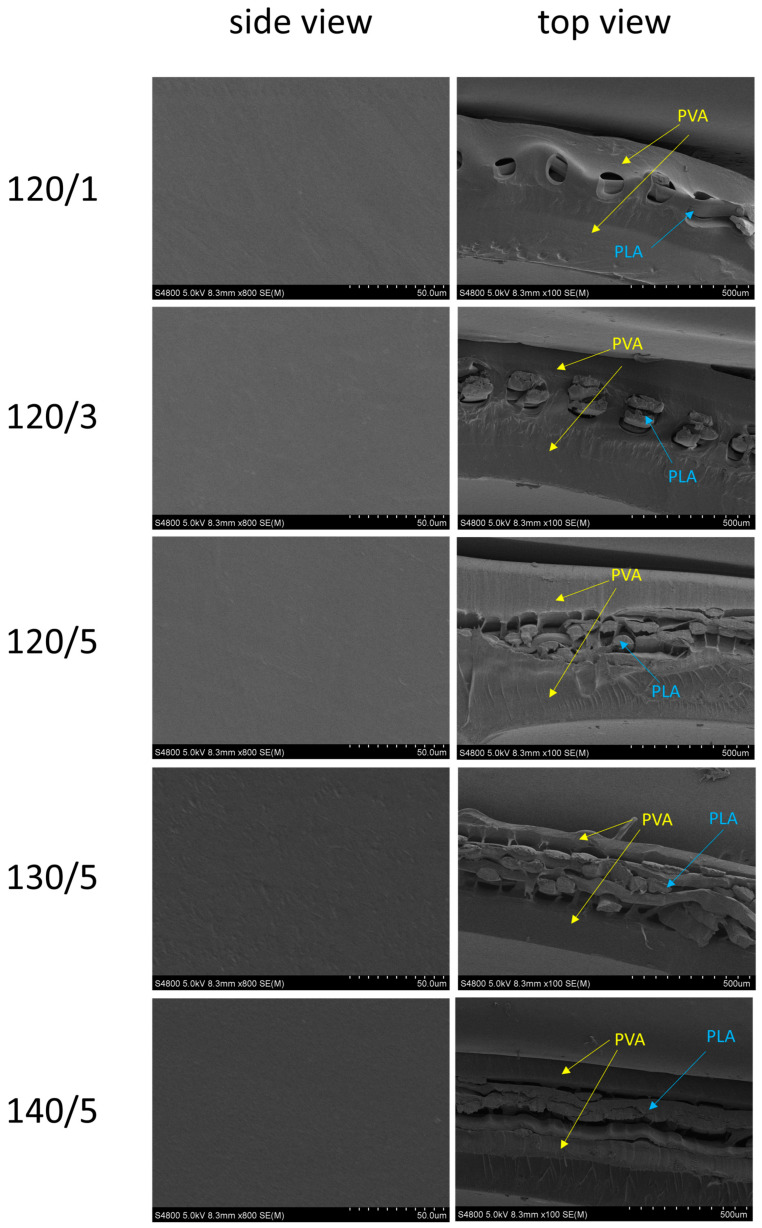
SEM image of the PVA/PLA hydrogels for different heat-treated temperatures and numbers of layers.

Figure 5 shows the SEM measurement results of the PVA/PLA hydrogel scaffolds as a function of the heat treatment temperature of PLA and the number of layers. The results are clear: The PLA mesh fabric was properly positioned inside the hydrogel. Cross-sectional measurements did not show any changes in appearance between samples. However, the thickness of the PLA mesh fabric tended to decrease with an increase in heat treatment temperature, especially for the sample with five layers. This led us to conclude that the PLA mesh fabric was thermally fused as the heat treatment temperature increased, leading to a decrease in strength.

### 2.6. MTT Assay

**Figure 6:**
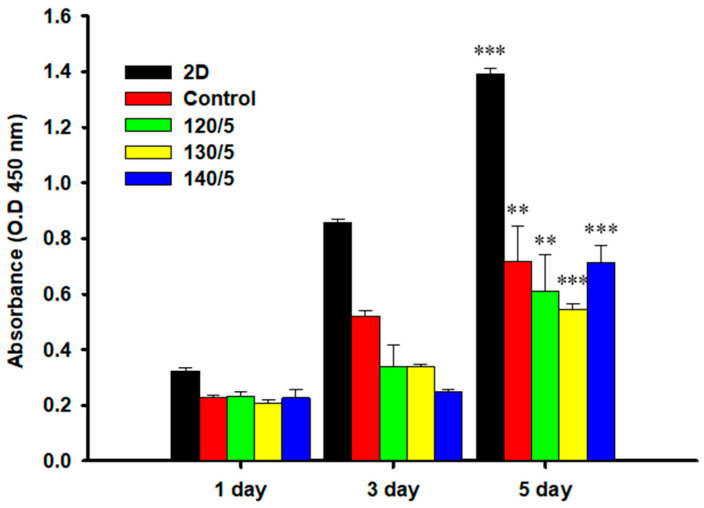
MTT assay test result of PVA/PLA hydrogels for different heat-treated temperatures in HaCaT cells. Significant differences between 1 day and 5 days are denoted by asterisks (** *p* < 0.01, *** *p* < 0.005).

Figure 6 shows the results of the biocompatibility and cell adhesion characterization of the PVA/PLA hydrogel. Comparative studies were conducted among two-dimensional control (2D), an unreinforced PVA hydrogel (control), and PVA/PLA hydrogels with embedded PLA mesh fabrics subjected to varying heat treatments of 120, 130, and 140 °C. The scaffolds displayed uniform initial cell adhesion, with no notable difference in proliferation rates between the PVA/PLA scaffolds and the control over the initial culture period. The marked proliferation increase on the fifth day versus the first day clearly demonstrates the nontoxic nature and compatibility of the hydrogels for cellular growth. It is clear that the optimal reinforcement effect was achieved at 120 °C, which also coincided with the highest recorded cell proliferation. This suggests a positive correlation between the thermal conditioning at this temperature and the scaffold’s performance. From the tensile strength and MTT assay results, we confirmed the improvement of tensile strength while maintaining cell viability at the heat treatment temperature of 120 °C. However, a comparison to the two-dimensional control revealed subdued growth, potentially ascribable to the limited duration of the study.

### 2.7. DAPI Staining

**Figure 7:**
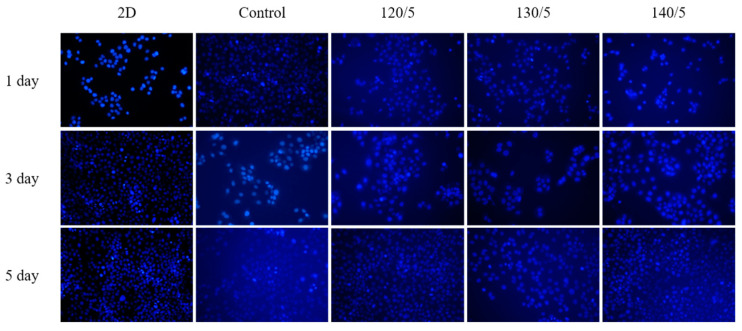
DAPI staining of PVA/PLA hydrogel scaffolds in HaCaT cell.

Figure 7 presents the DAPI staining of PVA/PLA hydrogel scaffolds in HaCaT cells, serving as a qualitative metric for biocompatibility and the absence of cytotoxicity. The consistent cell proliferation over a period of five days across all conditions underscores the biologically inert nature of the scaffolds. Notably, the reinforcement of PVA scaffolds with PLA mesh fabric, particularly following thermal treatment at 120 °C with five layers of mesh, demonstrated a favorable outcome in maintaining cellular viability and integrity. This enhancement is attributed to thermal conditioning, which likely induces a more favorable interface for cellular attachment and growth. Such findings are in alignment with the quantitative MTT assays, affirming the nontoxic profile and compatibility of the PLA mesh-reinforced scaffolds for potential tissue engineering applications. The comprehensive cellular response observed here emphasizes the potential of fine-tuning the structural and thermal properties of scaffold materials to optimize cellular interactions, thereby advancing the design of bioengineered constructs for regenerative medicine.

In this study, we utilized PLA mesh fabric to reinforce PVA hydrogels, markedly enhancing their mechanical strength and biocompatibility for tissue regeneration applications. The selection of PLA was driven by its cost-effectiveness, commercial availability, and established usage in products like tea bags. While other biodegradable polymers, such as PGA, PLGA, and PCL, offer benefits like tunable degradation rates, their higher costs and lack of commercial availability as mesh fabrics present significant limitations. These polymers possess unique properties that could be leveraged for specific biomedical applications.

Future research should investigate the use of various biodegradable polymer mesh fabrics to optimize the reinforcement of PVA hydrogels across a broad spectrum of biomedical applications. This study lays a crucial foundation for advancing the potential of biodegradable polymers in tissue engineering and regenerative medicine.

## 3. Conclusions

Our investigation into the synergistic effects of PLA mesh reinforcement within PVA hydrogel matrices, subjected to freeze–thaw physical crosslinking, revealed a complex interplay between the materials. While the inclusion of PLA mesh did not significantly alter the intrinsic gel content of the PVA/PLA composite, we observed a discernible decrease in swelling ratio with increased PLA mesh layers and heat treatment temperatures. This outcome is attributed to spatial constraints within the mold, where enhanced PLA layering and thermal conditions contribute to a void reduction on the mesh surface, thereby diminishing the swelling capacity.

Notably, additional PLA layers significantly enhanced mechanical integrity, with a peak increase of 36% in tensile strength observed in the quintuple-layered, 120 °C heat-treated scaffolds compared to the unenhanced controls. The heat treatment at 120 °C was clearly superior, displaying a 33% greater mechanical strength than scaffolds processed at 140 °C. This underscores the efficacy of optimal thermal processing in scaffold reinforcement strategies. The MTT assay results clearly showed that there were no significant differences in cellular proliferation rates between the PVA and PVA/PLA hydrogel. This demonstrates that the hydrogel is inherently noncytotoxic. Furthermore, the DAPI staining assays provide additional evidence of the hydrogel’s biocompatibility, with unimpeded cellular proliferation across the observation timeline.

Our findings highlight the importance of optimizing thermal processing conditions and PLA mesh layering to enhance the mechanical robustness of PVA hydrogels. Reinforced hydrogels are structurally robust, biocompatible, and hold significant potential for future biomaterial applications.

## 4. Materials and Methods

### 4.1. Materials and Chemicals

The PVA used in the experiments was grade F-17 from OCI Inc. Seoul, Republic of Korea, with a polymerization degree of 1700, a crystallinity of 98–99.5%, and a molecular weight of 74,800 g/mol. The PLA mesh fabric was manufactured by CircuLon (MA)^TM^ (Fairfield, CA, USA) as a 30-denier monofilament using 100% non-GMO PLA. The fabric specification was a plain weave with a density of 90 warp yarns/inch each, approximately 100 μm thick, 200/218 μm pore size, 60% porosity, and 25 g/m^2^ weight for tea bags provided by CORESHTECH Co. Ltd., Daegu, Republic of Korea.

### 4.2. Preparation of PVA/PLA Mesh Hydrogel Scaffold

**Figure 8:**
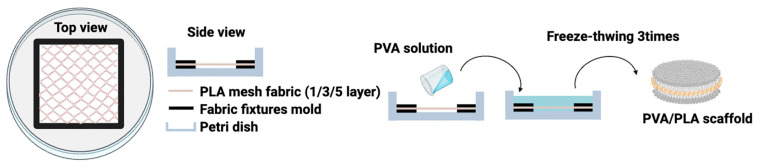
Schematic diagram of PVA/PLA scaffold manufacturing method.

The PLA mesh fabric for tea bags was cut into 10 cm × 10 cm pieces and then laminated with 1, 3, and 5 sheets at 120 °C (120/1, 120/3, 120/5) using a hot press to bond the fabric layer by layer to produce comparative samples based on the number of layers. Also, to produce comparative samples based on temperature, 5 sheets of fabric were laminated and hot-pressed at 120, 130, and 140 °C (120/5, 130/5, 140/5) to prepare samples. The fabrics were placed between two square silicone molds (4.5 cm × 4.5 cm) and fixed in the center of Petri dishes (90 cm × 15 mm). Next, to prepare the PVA hydrogel, 8 wt% PVA was added to DI water and dissolved at 100 °C for 5 h to prepare a mixed aqueous solution. The mixed aqueous solution was kept at the same temperature until the bubbles disappeared at 80 °C. The prepared PVA mixed aqueous solution was poured into the Petri dish with PLA mesh fabric installed and shaped. The polymer solution poured into the mold was left at room temperature for 3 h and then subjected to physical crosslinking using the freeze–thaw method and left at 40 °C for 48 h to prepare the hydrogel, as shown in Figure 8. The physical crosslinking using the freeze–thaw method was repeated three times by rapidly freezing the mold in a deep freezer at −58 °C and thawing at 40 °C. The prepared scaffolds were used by removing the molds.

### 4.3. Gel Content

To remove polymers that did not participate in the crosslinking reaction, the hydrogel was washed with stirring for 24 h at room temperature. The hydrogel was taken out after the washing process, the water on the gel surface was removed, and the gel was placed in an oven at 40 °C to dry for 24 h.

The gel content was expressed as a percentage by dividing the weight of the dried gel (W_d_) by the initial polymer weight (W_i_), as shown in Equation (1). The value was measured repeatedly (n = 10), and the mean value was calculated.

Gel content (%) = W_d_/W_i_ × 100 (1)

### 4.4. Swelling Ratio

The hydrogel was dried at 40 °C for 24 h and weighed (W_a_). The dried sample was immersed in distilled water at room temperature for 48 h and weighed (W_s_), and the swelling was calculated using Equation (2). The value was measured repeatedly (n = 10), and the mean value was calculated.

Swelling ratio (%) = W_s_/W_a_ × 100 (2)

### 4.5. Gel Strength Analysis

To investigate the physical properties of PVA/PLA mesh hydrogels, the tensile strength was measured at room temperature using AGS-X (Shimadzu Co., Kyoto, Japan). The thickness of the hydrogel specimen for tensile strength measurement was 30 mm, and the diameter was 10 mm. The cross-head speed of the strength measurement was 50 mm/min, and the value was measured 3 times and averaged.

### 4.6. FT-IR Analysis

An FT-IR analyzer, Nicolet iS20 (Thermo Scientific, Waltham, MA, USA), was used to measure the changes in the structure of the prepared hydrogels. These changes were analyzed in the wavelength range of 4000-600 cm^−1^ by taking four measurements with a resolution of 4 cm^−1^.

### 4.7. DSC Analysis

The thermal behavior of the hydrogel was measured using a differential scanning calorimeter DSC Q200 (Ta Instruments Co., New Castle, DE, USA). The temperature range was 40–250 °C in a nitrogen gas atmosphere, and the temperature rise was measured at 10 °C/min.

### 4.8. SEM Analysis

The cross-sectional and side-sectional views of the PVA/PLA hydrogel scaffolds were measured using an electron scanning microscope S-4800 (Hitachi, Tokyo, Japan).

### 4.9. MTT Assay

To determine the cell viability of the hydrogels, they were cut into squares with a diameter of 1 cm, disinfected with ethyl alcohol at concentrations of 20, 40, 60, 80, and 100% at 1 h intervals, and then sterilized in 70% ethyl alcohol for 24 h. The HaCaT cell culture medium was prepared using Dulbecco’s modified Eagle medium (DMEM, Gibco-BRL, Grand Island, NY, USA) containing 10% fetal bovine serum (FBS, Gibco BRL) and 1% antibiotics (penicillin–streptomycin, Gibco BRL), and then dispensed into cell culture flasks and cultured at 37 °C, 5% CO_2_ environment. The culture medium was changed daily. Monolayer cells formed in the incubator were detached using 0.05% trypsin–EDTA (Gibco BRL), centrifuged at 1000× *g* at 4 °C for 5 min, and then diluted with culture medium.

Cell viability was determined using the MTT assay. The cells (1 × 10^4^ cells/mL) were seeded in a 24-well plate and incubated for 24 h. Afterward, the cells were washed with phosphate-buffered saline (PBS), and MTT solution (5 mg/mL, Sigma-Aldrich, Burlington, MA, USA) was added to each well, followed by a 4 h incubation. The culture medium was discarded, and 100 μL dimethyl sulfoxide (Sigma-Aldrich) was added to dissolve formazan crystals. This mixture was then incubated for 20 min at room temperature. The absorbance was measured at a wavelength of 450 nm with a microplate reader (AMR-100, ALLSHENG Inc., Hangzhou, China), and the results were compared with those of the control at intervals of 1, 3, and 5 days).

### 4.10. DAPI Staining

DAPI staining was performed to observe morphological changes in cell nuclei. The cultured cells were washed with DMEM, followed by PBS, and then fixed in 10% formaldehyde for 15 min at room temperature. After fixation, the cells were washed with PBS and stained with 200 μL of DAPI solution for 10 min at room temperature. Following staining, the cells were washed again with PBS and analyzed under a fluorescence microscope (Eclipse Ts2R, Nikon Inc., Tokyo, Japan).

### 4.11. Statistical Analysis

Statistical analysis was performed using SAS software (version 9.4; SAS Institute, Cary, NC, USA). The results are expressed as means ± standard deviations. A *t*-test was employed to evaluate pairwise differences between the two groups, with a significance threshold of *p* < 0.05. Additionally, a one-way analysis of variance (ANOVA) with Duncan’s multiple-range test was conducted to identify significant differences between groups (*p* < 0.05).

## Figures and Tables

**Table 1:** Melting temperature for PVA/PLA scaffolds.

Hot Press Temperature (°C)/Number of PLA Layer	Melting Temperature (°C)	
	T_1_	T_2_
Control	-	222.01
120/5	158.88	221.30
130/5	158.28	220.59
140/5	155.39	211.97

## Data Availability

All data and materials are available upon request from the corresponding author. The data are not publicly available due to ongoing research using part of the data.

## References

[1] Capuana E., Lopresti F., Pavia F.C., Brucato V., La Carrubba V. (2021). Solution-Based Processing for Scaffold Fabrication in Tissue Engineering Applications: A Brief Review. Polymers.

[2] Manzini B.M., Machado L.M.R., Noritomi P.Y., da Silva J.V.L. (2021). Advances in Bone tissue engineering: A fundamental review. J. Biosci..

[3] Kim S.M., Kim K.M., Lee K.S., You C.K., Lee Y.K. (2011). Enhanced Strength of the Tissue Engineering Scaffold. J. Korean Res. Soc. Dent. Mater..

[4] Amanda J.R.W., Hilldore J., Lan S.K., Park C.J., Morgan A.W., Eurell J.A.C., Clark S.G., Wheeler M.B., Jamison R.D., Johnson A.J.W. (2007). The mechanical properties and osteoconductivity of hydroxyapatite bone scaffolds with multi-scale porosity. J. Biomater..

[5] Harley B.A., Leung J.H., Silva E.C.C.M., Gibson L.J. (2007). Mechanical characterization of collagen–glycosami-noglycan scaffolds. J. Acta Biomater..

[6] Paralikar S.A., Simonsen J., Lombardi J. (2008). Poly(vinyl alcohol)/Cellulose Nanocrystal Barrier Membrane. J. Membr. Sci..

[7] Figueiredo K.C.S., Alves T.L.M., Borges C.P. (2009). Poly(vinyl alcohol) Films Crosslinked by Glutaraldehyde Under Mild Conditions. J. Appl. Polym. Sci..

[8] Campos E., Coimbra P., Gill M.H. (2013). An Improved Method for Preparing Glutaraldehyde Cross-linked Chitosan-poly(vinyl alcohol) Microparticles. J. Polym. Bull..

[9] Beydaghi H., Javanbakht M., Badiei A. (2014). Cross-linked Poly(vinyl alcohol)/Sulfonated Nanoporous Silica Hybrid Membranes for Proton Exchange Membrane Fuel Cell. J. Nanostruct. Chem..

[10] Mansur H.S., Sadahira C.M., Souza A.N., Mansur A.A.P. (2008). FTIR Spectroscopy Characterization of Poly(vinyl alcohol) Hydrogel with Different Hydrolysis Degree and Chemically Crosslinked with Glutaraldehyde. J. Mater. Sci. Eng. C.

[11] Zhang Y., Zhu P.C., Edgren D. (2010). Crosslinking Reaction of Poly(vinyl alcohol) with Glyoxal. J. Polym. Res..

[12] Krumova M., López D., Benavente R., Mijangos C., Perena J.M. (2000). Effect of Crosslinking on the Mechanical and Thermal Properties of Poly(vinyl alcohol). J. Polym..

[13] Miyazaki T., Takeda Y., Akane S., Itou T., Hoshiko A., En K. (2010). Role of Boric Acid for a Poly(vinyl alcohol) Film as a Crosslinking Agent: Melting Behaviors of the Films with Boric Acid. J. Polym..

[14] Lim M., Kwon H., Kim D., Seo J., Han H., Khan S.B. (2015). Highly-enhanced Water Resistant and Oxygen Barrier Properties of Cross-linked Poly(vinyl alcohol) Hybrid Films for Packaging Application. J. Prog. Org. Coat..

[15] Yin Y., Li J., Liu Y., Li Z. (2005). Starch Crosslinked with Poly(vinyl alcohol) by Boric Acid. J. Appl. Polym. Sci..

[16] Kim G., Kim H., Kang H.J. (2011). Crosslinking Characteristic of Poly(vinyl alcohol) by Natural Dye. J. Polym..

[17] Jo S.Y., Lim Y.M., Youn M.H., Gwon H.J., Park J.S., Nho Y.C., Shin H.S. (2009). Fabrication and Characterization of PVA/CMC Hydrogels by Freezing-Thawing Technique and Gamma-Ray Irradiation. J. Polym..

[18] Sahoo S.K., Panda A.K., Labhasetwar V. (2005). Characterization of porous PLGA/PLA microparticles as a scaffold for three dimensional growth of breast cancer cells. J. Biomacromol..

[19] Liu X., Gao J., Cui X., Nie S., Wu X., Zhang L., Tang P., Liu J., Li M. (2023). Functionalized 3D-Printed PLA Biomimetic Scaffold for Repairing Critical-Size Bone Defects. J. Bioeng..

[20] Shi Z., Huang G., Li Z., Lou Z., Gong Z., Wang X., Li C., Wang B. (2023). A PLA-tPU based magnesium ion incorporated CSH/nHA bioactive porous composite scaffold for critical bone defect repair. J. Mater. Adv..

[21] Lee G., Lee H.M., Kim Y.H. (2019). Thermal and Mechanical Properties of Poly(L-lactic Acid) Films Plasticized with Propylene Carbonate. J. Polym..

[22] Da Silva D., Kaduri M., Poley M., Adir O., Krinsky N., Shainsky-Roitman J., Schroeder A. (2018). Biocompatibility, biodegradation and excretion of polylactic acid (PLA) in medical implants and theranostic systems. J. Chem. Eng..

[23] Cha M., Jin Y.Z., Park J.W., Lee K.M., Han S.H., Choi B.S., Lee J.H. (2021). Three-dimensional printed polylactic acid scaffold integrated with BMP-2 laden hydrogel for precise bone regeneration. J. Biomater. Res..

[24] Tyler B., Gullotti D., Mangraviti A., Utsuki T., Brem H. (2016). Polylactic acid (PLA) controlled delivery carriers for biomedical applications. J. Adv. Drug Deliv. Rev..

[25] Nasr M., Awad G.A., Mansour S., Al Shamy A., Mortada N.D. (2013). Hydrophilic versus hydrophobic porogens for engineering of poly(lactide-co-glycolide) microparticles containing risedronate sodium. J. Pharm. Dev. Technol..

[26] Seo Y.H., Zo S.M., Han S.S., Oh T.H. (2023). Study on Mechanical Properties and Biocompatibility of Scaffold Made of using PLA Mesh Fabric. J. Text. Sci. Eng..

